# Anatomical identification of a corticocortical top-down recipient inhibitory circuitry by enhancer-restricted transsynaptic tracing

**DOI:** 10.3389/fncir.2023.1245097

**Published:** 2023-08-30

**Authors:** Yusuke Atsumi, Yasuhiro Oisi, Maya Odagawa, Chie Matsubara, Yoshihito Saito, Hiroyuki Uwamori, Kenta Kobayashi, Shigeki Kato, Kazuto Kobayashi, Masanori Murayama

**Affiliations:** ^1^Laboratory for Haptic Perception and Cognitive Physiology, RIKEN Center for Brain Science, Saitama, Japan; ^2^Department of Life Science and Technology, School of Life Sciences and Technology, Tokyo Institute of Technology, Tokyo, Japan; ^3^Department of Biology, Graduate School of Science, Kobe University, Kobe-shi, Japan; ^4^Section of Viral Vector Development, National Institute for Physiological Sciences, Okazaki-shi, Japan; ^5^Department of Molecular Genetics, Institute of Biomedical Sciences, Fukushima Medical University School of Medicine, Fukushima, Japan

**Keywords:** anterograde transsynaptic tracing, enhancer-based viral vectors, inhibitory neurons, corticocortical circuit, top-down input

## Abstract

Despite the importance of postsynaptic inhibitory circuitry targeted by mid/long-range projections (e.g., top-down projections) in cognitive functions, its anatomical properties, such as laminar profile and neuron type, are poorly understood owing to the lack of efficient tracing methods. To this end, we developed a method that combines conventional adeno-associated virus (AAV)-mediated transsynaptic tracing with a distal-less homeobox (Dlx) enhancer-restricted expression system to label postsynaptic inhibitory neurons. We called this method “Dlx enhancer-restricted Interneuron-SpECific transsynaptic Tracing” (DISECT). We applied DISECT to a top-down corticocortical circuit from the secondary motor cortex (M2) to the primary somatosensory cortex (S1) in wild-type mice. First, we injected AAV1-Cre into the M2, which enabled Cre recombinase expression in M2-input recipient S1 neurons. Second, we injected AAV1-hDlx-flex-green fluorescent protein (GFP) into the S1 to transduce GFP into the postsynaptic inhibitory neurons in a Cre-dependent manner. We succeeded in exclusively labeling the recipient inhibitory neurons in the S1. Laminar profile analysis of the neurons labeled via DISECT indicated that the M2-input recipient inhibitory neurons were distributed in the superficial and deep layers of the S1. This laminar distribution was aligned with the laminar density of axons projecting from the M2. We further classified the labeled neuron types using immunohistochemistry and *in situ* hybridization. This *post hoc* classification revealed that the dominant top-down M2-input recipient neuron types were somatostatin-expressing neurons in the superficial layers and parvalbumin-expressing neurons in the deep layers. These results demonstrate that DISECT enables the investigation of multiple anatomical properties of the postsynaptic inhibitory circuitry.

## 1. Introduction

Inhibitory circuitries in the neocortex mediate crucial modulations of cognitive processes in various behavioral contexts (Letzkus et al., 2011; Fu et al., 2014; Lovett-Barron and Losonczy, 2014; Wester and McBain, 2014; Kato et al., 2015; Makino and Komiyama, 2015; Kuchibhotla et al., 2016; Tremblay et al., 2016; Attinger et al., 2017; Muñoz et al., 2017; Abs et al., 2018; Khan et al., 2018; Kirchberger et al., 2021). The circuits are regulated not only by intracortical projections but also by mid/long-range projections from other cortical (Palmer et al., 2012; Lee et al., 2013; Yang et al., 2013; Schneider et al., 2014; Zhang et al., 2014; Ibrahim et al., 2016; Naskar et al., 2021; Shen et al., 2022) and subcortical regions (Ji et al., 2016; Yu et al., 2016; Audette et al., 2018, 2019; Takesian et al., 2018; Sermet et al., 2019; Fang et al., 2020; Naskar et al., 2021). These projections selectively innervate specific subtypes of postsynaptic inhibitory circuitries that modulate neural activity in response to contextual demands, such as attentional modulation (Zhang et al., 2014) and sensorimotor integration (Lee et al., 2013; Schneider et al., 2014). Therefore, identifying the projection-specific properties of the postsynaptic inhibitory circuitry, such as the laminar profiles and neuron types, allows us to estimate the functional roles of the projections and the operational mechanisms of the circuits. However, a comprehensive understanding of the postsynaptic inhibitory circuitry has not been achieved.

Retrograde transsynaptic tracings by rabies virus-based tools have been widely used to label presynaptic circuitries (Wickersham et al., 2007; Wall et al., 2010). Although these tracings are also used to estimate postsynaptic circuitries (Ma et al., 2021), interpretations of anatomical results need to be treated with care because of indirect tracing of postsynaptic neurons. An anterograde approach using a combined method of neurophysiological recording and optogenetic stimulation, channelrhodopsin-2 (ChR2)-assisted circuit mapping (CRACM), has been used to directly assess the relationships between pre-and postsynaptic neurons (Petreanu et al., 2007; Yang et al., 2013); however, the number of recorded postsynaptic neurons is limited (a few neurons per recording site in general), which makes the experiments inefficient.

**FIGURE 1:**
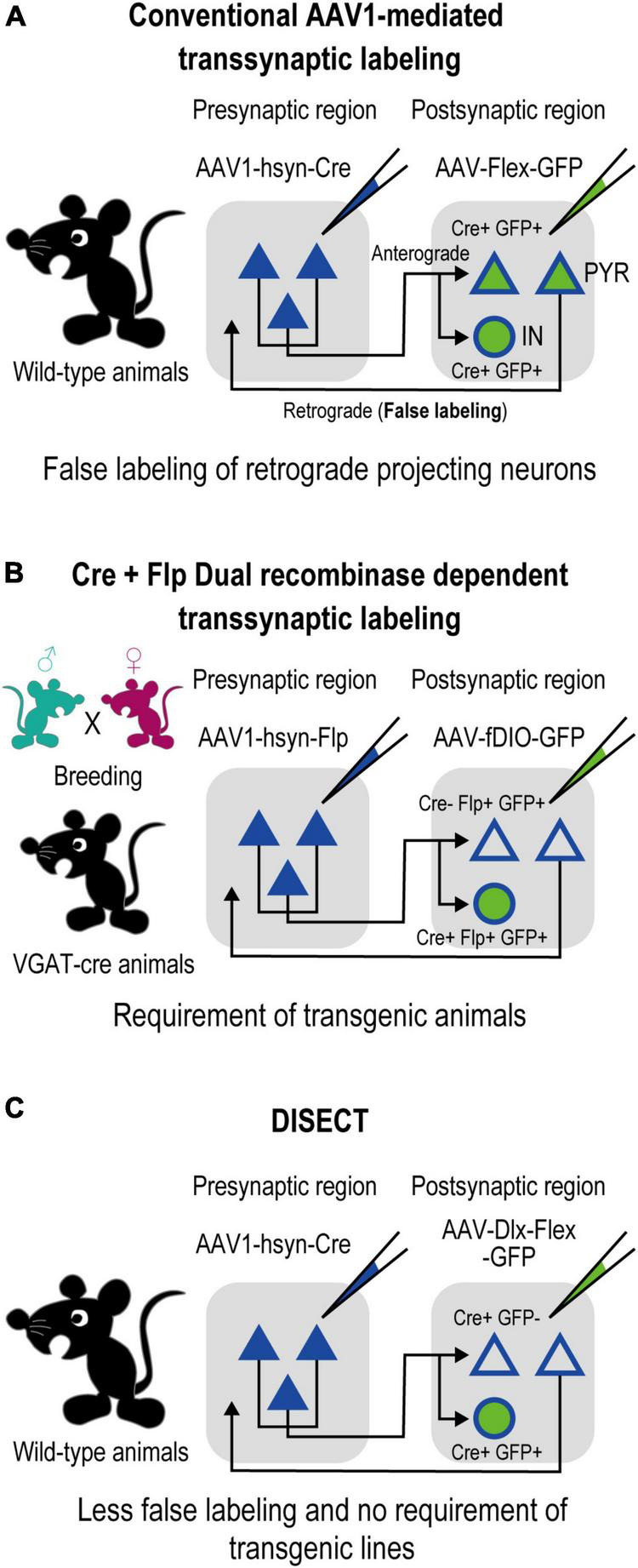
Comparison of DISECT with the AAV1-mediated anterograde transsynaptic tracing method. **(A)** Schematic of the conventional AAV1-mediated method shown in a previous study (Zingg et al., 2017). Injections of AAV1-Cre into the presynaptic region enable *trans-*synaptical spread of infection of the vector, allowing Cre-dependent transduction of genes at the postsynaptic neurons. However, the subtype-independent spread induces gene expression at excitatory neurons, which disturbs the *post hoc* analysis of subtype specificity of inhibitory neurons and decreases the interpretability of the results (see also Section “4. Discussion”). **(B)** Schematic of a complex use of the AAV1-mediated method shown in a previous study (Zingg et al., 2017). Using transgenic animals (such as VGAT-Cre mice, which express Cre specifically in inhibitory neurons) allows us to distinguish excitatory projecting neurons from postsynaptic neurons, increasing interpretability. However, this method requires complex procedures, such as preparation of transgenic animals. **(C)** Schematic of DISECT developed in this study. By injecting a Cre-inducible Dlx enhancer-specific AAV vector into the postsynaptic region, transduction can be restricted only to the postsynaptic inhibitory neurons with simple procedures, ensuring simplified efficient tracing with high interpretability in wild-type animals.

The anterograde transsynaptic tracing method has also been conventionally used for the direct investigation of postsynaptic neuronal circuitries (Yoshihara et al., 1999; Kissa et al., 2002; Lo and Anderson, 2011). Recent studies have reported that adeno-associated virus serotype 1 (AAV1) can spread anterogradely via transneuronal transport (Zingg et al., 2017, 2020), suggesting that AAV1 is a promising anterograde transsynaptic viral vector. When presynaptic neurons are infected with AAV1-Cre, the infection of the vector *trans-*synaptically spreads to postsynaptic neurons, allowing for the characterization of postsynaptic neurons with Cre-dependent expression of fluorescent proteins, such as GFP (Figure 1A). This method labels a mixed population of excitatory and inhibitory neurons. However, in corticocortical reciprocal circuits, the labeled population is contaminated by a large fraction of falsely labeled retrogradely projecting excitatory neurons that project back to their presynaptic region because of a capacity for retrograde transport of AAV1-Cre. As a result, the subsequent analysis of subtype specificity, such as calculation of the fraction of labeled neurons coexpressing neurochemical markers, was disturbed. To distinguish excitatory projecting neurons from postsynaptic neurons, an additional orthogonal intersectional system such as Flp/fDIO (Figure 1B) can be used. However, the orthogonal intersectional systems also require time-consuming procedures, such as the preparation of transgenic animals.

Herein, we propose a simplified method using AAV1-assisted enhancer-restricted expression techniques to achieve specific gene expression in postsynaptic inhibitory neurons, which could replace the additional use of intersectional systems. The distal-less homeobox (Dlx) enhancer restricts gene expression exclusively in GABAergic inhibitory neurons (Dimidschstein et al., 2016). By combining this method with the conventional AAV1-mediated transsynaptic tracing, we developed “Dlx+ Interneuron-Specific Transsynaptic tracing” (abbreviated as DISECT), a method that allows more efficient transsynaptic tracing of postsynaptic inhibitory neurons (Figure 1C). Compared with previous methods, DISECT largely simplifies the procedures for labeling postsynaptic inhibitory neurons by eliminating the need for dual intersectional systems or preparation of transgenic animals. In this study, we used DISECT to investigate the characteristics of top-down input-recipient inhibitory neurons. We previously reported that the secondary motor cortex (M2) projects top-down cortical inputs to the primary somatosensory cortex (S1). These projections play a crucial role in accurate somatosensory perception (Manita et al., 2015) and perceptual memory consolidation (Miyamoto et al., 2016). Herein, we report the laminar profiles and neuron subtypes of top-down recipient inhibitory S1 neurons.

## 2. Materials and methods

### 2.1. Animals

All animal experiments were performed in accordance with institutional guidelines and were approved by the Animal Experiment Committee at RIKEN. Male wild-type mice (10–13 weeks old) (C57BL/6JJmsSlc; Japan SLC, Shizuoka, Japan) were used in this study. In all experiments, the mice were housed in a 12 h-light/12 h-dark light cycle environment with *ad libitum* access to food and water.

### 2.2. AAV vector preparation

The following plasmids were obtained from Addgene (Addgene, Watertown, MA, USA): pAAV-CaMKII-ChrimsonR-tdTomato (cat. #9923; RRID:Addgene_99231) and pAAV-hDlx-Flex-green fluorescent protein (GFP)-Fishell_6 (cat. #83895; RRID:Addgene_83895). AAV was produced as previously described (Kobayashi et al., 2016; Kato et al., 2018). The following AAV vectors were obtained from Addgene: AAV-hSyn-Cre-WPRE-hGH (cat. #105553-AAV1; RRID:Addgene_105553) and AAV-hDlx-Flex-GFP-Fishell_6 (cat. #83895-AAV; RRID:Addgene_83895).

### 2.3. Stereotaxic injections of CTB and AAV vectors for retrograde and anterograde tracing, DISECT

The animals were anesthetized with isoflurane (2–3% for induction and 1.2% for maintenance) using anesthesia equipment (AN-487-0T, Shinano, Japan). Stereotaxic injections were administered to deliver Cholera Toxin Subunit B (CTB) conjugate 647 (Cat#C34778, Molecular Probes, USA) or AAV to specific cortical areas. A craniotomy was performed above the injection site and CTB or AAV was injected (10 nL/min) via a pulled glass pipette (Cat#BR708744, BRAND, USA). To mark the injection of AAVrg-hSyn-Cre-WPRE-hGH, we co-injected Hoechst 33342 (Cat#19172-51, NACALAI TESQUE, Japan), because fluorescent probes were not present in the virus itself (Mukherjee et al., 2020). The coordinates, volumes, and titers were as follows: left M2 [anteroposterior from bregma (AP), + 2.19 mm; mediolateral from the midline (ML), 0.6 mm; dorsoventral from the cortical surface (DV), 3 depth in 0.2–0.8 mm, 600 nL, 5.50 × 10^12^ GC/mL for AAV1-CaMKII-ChrimsonR injections, 100 nL, 1.00 × 10^13^ GC/mL for AAV1-hsyn-Cre-hGH-WPRE], left S1 (AP, −0.75 mm; ML, 1.70 mm; DV, 0.2–0.7 mm, 150 nL, 0.5%, for CTB injections, 200 nL, 3.33 × 10^12^ GC/mL for AAV1-hdlx-flex-GFP). For the experiment using DISECT, the AAV1-hdlx-flex-GFP that was produced in our own laboratory was used in four of five mice and those obtained from Addgene was used in one of five mice. We confirmed that the VGAT-expression, the laminar distribution, and the expression of neurochemical markers were similar between neurons labeled by both of the two vectors.

### 2.4. Tissue preparation

Mice were deeply anesthetized using intraperitoneal injection of urethane and perfused transcardially with 20 mL of Hanks’ Balanced Salt Solution (HBSS; 14025076, Life Technologies, USA) supplemented with heparin (10 units/mL), followed by perfusion with 4% formaldehyde in 0.1 M phosphate buffer (PB; pH 7.4) and postfixation in the same fixative for 16–20 h at 4°C. For subsequent *in situ* hybridization experiments, the brain blocks were cut into 40-μm-thick coronal sections using a freezing microtome (ROM-380, Yamato, Japan) after cryoprotection with 30% sucrose in PB. For the other experiments, the brain blocks were cut into 80-μm-thick coronal sections on a vibratome (VTS1200S, LEICA, Germany).

### 2.5. Immunofluorescence labeling

Free-floating sections were incubated in a blocking solution [2% normal goat serum (NGS) and 0.3% Triton X-100 in phosphate-buffered saline (PBST)] at room temperature for 1 h, followed by incubation with 1/2000-diluted rabbit anti-Parvalbumin IgG (Cat#PV27, Swant, Switzerland, RRID:AB_2631173) and 1/500-diluted goat anti-Somatostatin IgG (Cat#sc-7819, Santa Cruz, RRID:AB_2302603) in 2% NGS overnight at 4°C. After washing for 10 min with PBST three times, the sections were incubated with donkey anti-rabbit secondary antibodies conjugated to 1/300-diluted Alexa Fluor Plus 568 (Cat#A10042, Molecular Probes, USA, RRID:AB_2534017) and donkey anti-goat secondary antibodies conjugated to 1/300-diluted Alexa Fluor Plus 647 (Cat#A32849, Molecular Probes, USA, RRID:AB_2762840) in 2% NGS for 2 h at room temperature. Subsequently, the sections were rinsed with PBS and mounted on a cover glass using Fluoromount (Cat#K048, Diagnostic BioSystems). For experiments requiring laminar identification, the sections were incubated with 1/500-diluted NeuroTrace™ 435/455 Blue Fluorescent Nissl Stain (Cat#21479; Thermo Fisher Scientific) overnight. To identify vasoactive intestinal peptide (VIP) and neuropeptide-Y (NPY) neurons, 1/300-diluted anti-VIP (Cat#20077, ImmunoStar, RRID:AB_572270) and 1/50-diluted anti-NPY (Cat#NMD-MSFR104610, Nittobo) were also used. However, we could not obtain results with a good signal-to-noise ratio sufficient for subsequent automatic detection of somata. Therefore, we analyzed the images obtained by the fluorescent *in situ* hybridization staining method.

### 2.6. FISH

Fluorescent in situ hybridization (FISH) was performed using the RNAscope system (Advanced Cell Diagnostic, Newark, CA, USA). Two 40 μm coronal sections of the S1 area were processed according to the RNAscope Multiplex Fluorescent Reagent Kit v2 User Manual (Cat#323100; ACD). RNAscope^®^ Target Probes against VGAT (Mm-Slc32a1-C2 Mouse, Cat#416631-c2), VIP (Mm-Vip-C2 Mouse, Cat#415961-c2), or NPY (Mm-Npy-C2 Mouse, Cat#313321-c2) were used. After the final wash buffer, the sections were washed with 0.3% PBST for 20 min. Subsequently, sections were incubated with 1/300-diluted chicken anti-GFP IgY (Cat#13970, Abcam) diluted in 2% NGS overnight at 4°C. After washing three times for 5 min with PBST, the sections were incubated with 1/250-diluted goat anti-chicken secondary antibodies conjugated to Alexa Fluor Plus 488 (Cat#A32931, Thermo Fisher Scientific) in 2% NGS for 2 h at room temperature. The sections were washed once with PBST for 5 min, treated with 1/1000 diluted 4′,6-diamidino-2-phenylindole (DAPI; Cat#D9542, Sigma-Aldrich) in PBS for 10 min, rinsed with PBS, then mounted on a cover glass using Fluoromount.

### 2.7. Microscopy for brains and tissue sections

**FIGURE 2:**
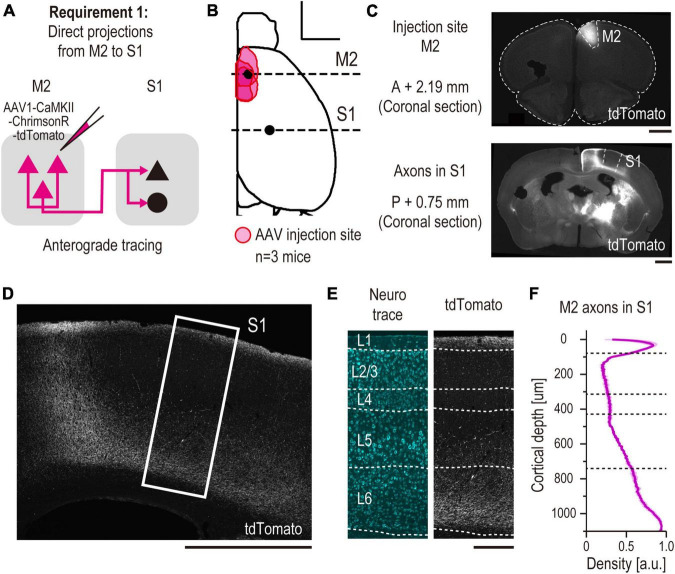
Anatomical demonstration of a direct long-range projection from M2 to S1 using anterograde tracing (validation for requirement 1). **(A)** Experimental schematic of anterograde tracing. **(B)** All the injection sites of AAV are projected onto the Allen Mouse Brain Common Coordinate (See Section “2. Materials and methods”). The red area indicates the injection site in each mouse (*n* = 3 mice, scale bar, 2,000 μm). The horizontal dashed line indicates the coordinate of observed coronal brain sections containing M2 [AP, + 2.19 mm, panel **(C)** top] and S1 [AP, –0.75 mm, panel **(C)** bottom] regions. **(C)** Representative images of coronal sections. (top) A section containing an injection site in M2. (bottom) A section containing S1 (scale bar, 1,000 μm). **(D)** Enlarged representative images of tdTomato + axons from M2 in S1 (scale bar, 1,000 μm). The white rectangle indicates the cropped S1 region shown in panel **(E)**. **(E)** Laminar-specific axonal pattern in S1. White dashed line indicates the layer division determined by Neurotrace staining. (left) Neurotrace (cyan). (right) tdTomato (gray) (scale bar, 250 μm). **(F)** Normalized density of M2 axons (arbitrary units, a.u.) versus distance from pia (in micrometers, *n* = 3 mice).

**FIGURE 3:**
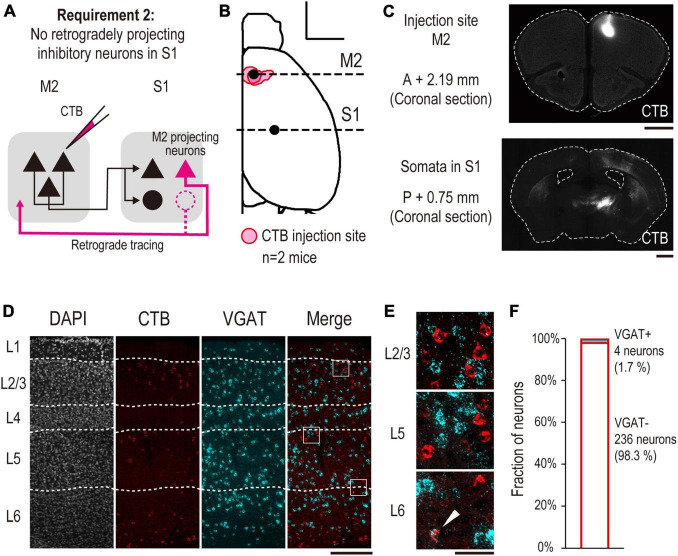
Anatomical demonstration of no S1 INs projecting back to M2 using retrograde tracing (validation for requirement 2). **(A)** Experimental schematic of retrograde tracing. **(B)** All injection sites of CTB are projected onto the Allen Mouse Brain Common Coordinate. The red area indicates the injection site in each mouse (*n* = 2 mice, scale bar, 2,000 μm). The horizontal dashed line indicates the coordinate of observed coronal brain sections containing M2 (AP, +2.19 mm, Figure 2C top) and S1 [AP, –0.75 mm, panel **(C)** bottom] regions. **(C)** Representative images of coronal sections. (top) A section containing an injection site in M2. (bottom) A section containing S1 (scale bar, 1,000 μm). **(D)** A representative image of laminar-specific distribution of M2 projecting S1 neurons labeled by CTB. A section was treated by FISH to visualize VGAT mRNA expression. White dashed line indicates the layer division determined by Neurotrace staining. From left to right, Neurotrace (gray), CTB (red), VGAT mRNA (cyan), merged image of CTB and VGAT mRNA (scale bar, 250 μm). The white squares indicate the cropped regions shown in panel **(E)**. **(E)** From top to bottom, enlarged images of layers 2/3, 5, and 6 of the merged image shown in panel **(D)**, respectively. The white arrowhead indicates a neuron expressing VGAT mRNA, which is also labeled by CTB (scale bar, 50 μm). **(F)** Fractions of VGAT + (cyan, *n* = 4/240 neurons, 1.7%) and VGAT- (white, *n* = 236/240 neurons, 98.3%) neurons labeled by CTB, respectively.

**FIGURE 4:**
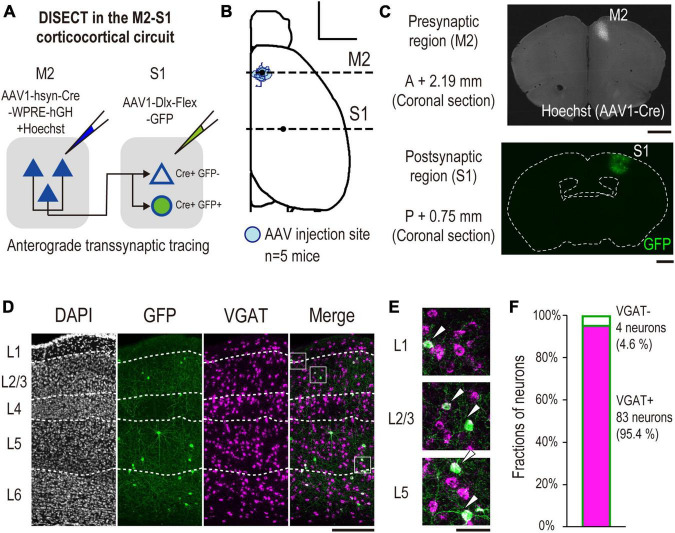
Validation of VGAT mRNA expression in labeled S1 neurons by DISECT. **(A)** Experimental schematics of DISECT. **(B)** All injection sites of AAV1-hsyn-Cre/hoechst33342 are projected onto the Allen Mouse Brain Common Coordinate. The red area indicates the injection site in each mouse (*n* = 5 mice, scale bar, 2,000 μm). The horizontal dashed line indicates the coordinate of observed coronal brain sections containing M2 (AP, +2.19 mm, Figure 2C top) and S1 (AP, –0.75 mm, Figure 3C bottom) regions. **(C)** Representative images of coronal sections. (top) A section containing an injection site in M2. (bottom) A section containing S1 (scale bar, 1,000 μm). **(D)** Representative images of laminar-specific distribution of S1 neurons labeled by DISECT. A section was treated by FISH to visualize VGAT mRNA expression. White dashed line indicates the layer division determined by DAPI staining. From left to right, DAPI (gray), GFP (green), VGAT mRNA (magenta), merged image of GFP and VGAT mRNA (scale bar, 250 μm). The white squares indicate the cropped regions shown in panel **(E)**. **(E)** From top to bottom, enlarged images of layers 1, 2/3, and 5 of the merged image shown in panel **(D)**, respectively. The white arrowhead indicates neurons coexpressing GFP and VGAT mRNA (scale bar, 50 μm). **(F)** Fractions of GFP+, VGAT+ (cyan, *n* = 83/87 neurons, 2 mice, 95.4%), and VGAT- (white, *n* = 4/87 neurons, 2 mice, 4.6%) neurons labeled by GFP, respectively.

**FIGURE 5:**
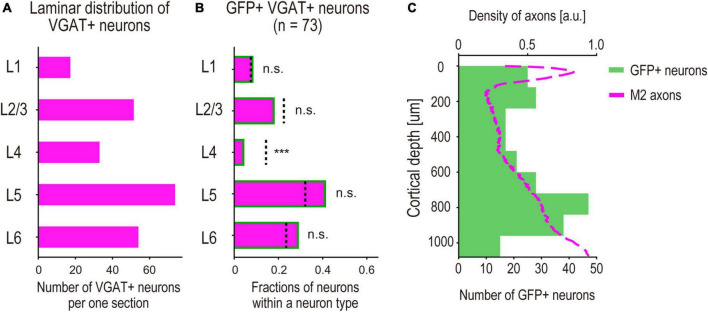
Laminar profiles of M2-input recipient S1 INs. **(A)** Average of the density of observed VGAT + neurons in each cortical layer per section. **(B)** Fractions of GFP + VGAT + neurons (*n* = 73, 2 mice, M2-input recipient INs) in each cortical layer. (bar graph) Fractions of the neurons in each cortical layer. (dashed line) Expected fractions of VGAT + neurons in each cortical layer, which are obtained by calculating the neuronal density ratio of VGAT + neurons from the result shown in panel **(A)**. Significance of fractions tested using binomial test: *** indicates *p* < 0.001, n.s. indicates *p* > 0.05. **(C)** Laminar distribution of M2-input recipient INs. (green bar graph) The counted neurons per section of 120 μm depth versus distance from pia (*n* = 236 neurons, 5 mice) (magenta line). Normalized density of M2 axons versus distance from pia shown in Figure 2F (arbitrary units, a.u.).

To confirm the injection sites or the labeled tissues of the brain blocks and fluorescence-labeled sections, the specimens were observed under an inverted microscope (IX83P2, Olympus) equipped with a 1.25 × /0.04 N.A. air objective (Cat#PLAPON1.25X, Olympus), as shown in Figures 2B, 3B, 4B, or a 4 × /0.16 N.A. air objective (Cat# UPLXAPO4X, Olympus), as shown in Figures 2C, 3C, 4C. To acquire images for subsequent analysis of coexpressing neurons labeled with CTB, GFP, or each neurochemical marker, the sections were observed under a confocal laser scanning microscope (FV3000RS, Olympus) with a 20 × /0.80 N.A. air objective (Cat # UPLXAPO20X, Olympus) (Figures 2D, E, 3D, E, 4D, E, 5A).

### 2.8. Data analysis

All analyses were performed using custom-built programs in Python or Fiji/ImageJ 1.53c (Schindelin et al., 2012).

#### 2.8.1. Quantification of injection site locations

A dorsal image of the dissected mouse brain was acquired, and the coordinates of the injection sites were normalized using image-processing procedures and brain normalization. First, the images were represented as binary images, and the contours of the hemisphere were acquired using image-processing functions implemented in the Python OpenCV library. Second, a dorsal view of the contours was acquired from the Allen Mouse Brain Common Coordinate Framework (Wang et al., 2020). Third, at least 10 feature points were defined on the actual and normalized hemispheres; the feature points were defined as “the endpoints of the lateral contour of the cortex, points that divide the contour into four segments, and points defined by projecting all points on the contour onto the midline.” Next, a non-rigid transformation was implemented in the Python OpenCV library (ThinPlateSplineShapeTransformer) that matched the feature points of the actual hemisphere to the normalized one. Finally, the transformed image was represented as a binary image, and the injection site was defined at an intensity above the threshold (Figures 2B, 3B, 4B).

#### 2.8.2. Determination of the layer division of cortex

**FIGURE 6:**
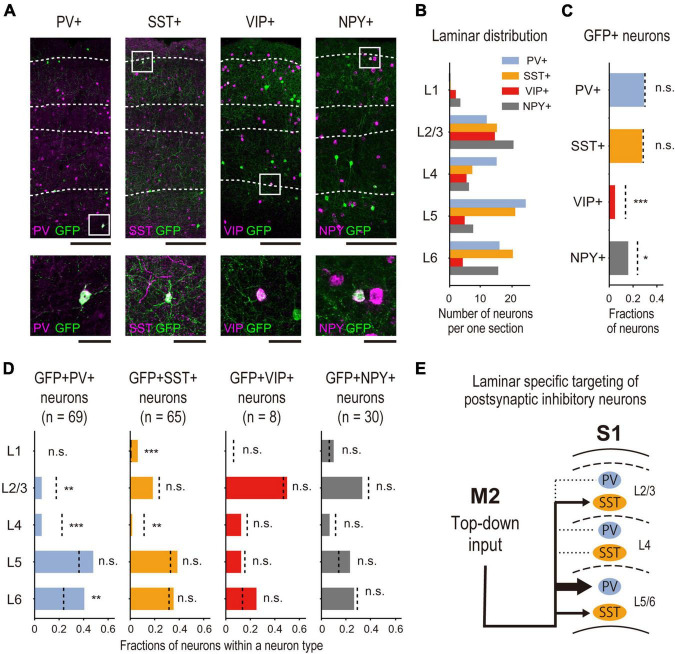
Subtype specificity of M2-input recipient S1 INs. **(A)** Representative images of laminar-specific distribution of S1 neurons labeled by DISECT. Each section is treated by IHC (PV, SST) or FISH (VIP, NPY) to visualize the expression of each neurochemical marker. The white dashed line indicates the layer division determined by Neurotrace or DAPI staining. From left to right, PV, SST, VIP, and NPY (scale bar, top: 250 μm, bottom: 50 μm). Green indicates GFP, and magenta indicates the expression of each neurochemical marker. The white squares indicate the cropped regions shown at the bottom. **(B)** Laminar distribution of the average density of neurons expressing each neurochemical marker in each cortical layer (*n* = 5 mice). **(C)** Fractions of GFP + X + neurons (M2-input recipient INs, X stands for each neurochemical marker. PV: *n* = 69 neurons, 5 mice, SST: *n* = 65 neurons, 5 mice, VIP: *n* = 8 neurons, 5 mice, NPY: *n* = 30, 5 mice) expressing each neurochemical marker among total GFP + neurons. (bar graph) Fractions of the neurons. (dashed line) Expected fractions of X + neurons, which are obtained by calculating the relative total neuronal density ratio of X + neurons **(A)** to VGAT + neurons (Figure 5A) from the result shown in Figure 5A. Significance of fractions tested using the binomial test: *** indicates *p* < 0.001, * indicates *p* < 0.05, n.s. indicates *p* > 0.05. **(D)** The same analysis as shown in Figure 5B is performed among neurons expressing each marker in each cortical layer. (bar graph) The fractions of the neurons in each cortical layer. (dashed line) The expected fractions of X + neurons in each cortical layer. Significance of fractions tested using the binomial test: *** indicates *p* < 0.001, ** indicates *p* < 0.01, n.s. indicates *p* > 0.05. **(E)** Schematic summary of identified M2 top-down input recipient inhibitory circuitry in S1. M2 top-down input preferentially targets PV + or SST + INs rather than VIP + or NPY + . Particularly, they preferentially target PV + INs in deep layers rather than superficial layers.

Images of coronal sections containing the S1 regions were acquired, which were processed using Neurotrace or DAPI staining (Figures 2E, F, 3D, 4D, 6A). The boundaries between layers were defined based on their cytoarchitecture.

#### 2.8.3. Laminar-density analysis of axons from the M2

From the image of the S1 area, a 500-μm wide region containing all cortical layers was cropped. Cortical depths of different samples were normalized to the distance from the pia to the white matter. The fluorescence intensity of the region was normalized to pixels of maximum intensity. To estimate the laminar density of axons, the region was subdivided into approximately 110 subdivisions of 500 × 10 μm^2^ each, and the total intensity was measured for each subdivision and plotted against the normalized cortical depth (Figures 2F, 4F).

#### 2.8.4. Detection of marker-expressing neurons and marker-coexpressing neurons

A region with 500-μm width was cropped for the following analysis using custom-built programs in ImageJ macro. The image was processed using the following procedure (Figures 3F, 4F, 5B, 6B–D). (i) To segment the contours of somata, the image was filtered by median filter. Then, the background signal was subtracted from the image. When processing the images of the sections stained with somatostatin (SST) antibody, the image was subsequently filtered with a Gaussian filter and the background was subtracted again because labeled axons and dendrites are often misidentified as soma. (ii) The image was represented as a binary image by thresholding all pixels. (iii) Regions of interest (ROIs) were defined by the functions of “analyze particles.” (iv) False positive particles, whose morphology was not similar to that of neurons were manually removed from the datasets. The size of some of the particles was significantly smaller than the pixel size threshold of the “analyzed particles” function, despite their neuron-like morphology. Because these particles were not detected due to their size, they were manually classified as true positive neurons. The filter parameters were changed depending on the targets (CTB, GFP, VGAT, PV, SST, VIP, or NPY). After defining the ROIs, we validated the overlap between CTB or GFP and the neurochemical markers. ROIs that included overlapping pixels were defined as “coexpressing neurons.”

#### 2.8.5. Statistical comparison of fractions of subtypes of inhibitory neurons using binomial tests

Two-sided binomial tests were performed to determine the statistical significance of fractions of neurons. Binomial tests required the “tested” and “expected” fractions for comparison. In the entire population analysis of all layers (Figure 6C), the tested fractions were defined as the neuronal density ratio of GFP+ neurons coexpressing neurochemical markers to VGAT mRNA-expressing neurons, and the expected fractions were defined as the ratio of neurons expressing the markers to VGAT mRNA-expressing neurons. In the population analysis of each cortical layer (Figures 5B, 6D), the tested fractions were defined as the neuronal density ratio of GFP+ neurons coexpressing the markers in each cortical layer to the total GFP+ neurons, and the expected fractions were defined as the ratio of neurons expressing all markers in each cortical layer to the total number of marker-expressing neurons. The neuronal density was obtained by calculating the average number of neurons in all the samples used for the quantitative analysis.

## 3. Results

### 3.1. Anatomical requirements for applying DISECT

Dlx enhancer-restricted Interneuron-SpECific transsynaptic Tracing is a simplified, two-step viral injection approach. In the first step, injection of AAV1-Cre into the presynaptic region enabled Cre-induced gene expression in recipient postsynaptic neurons. In the second step, injection of a Cre-inducible Dlx enhancer-specific AAV vector restricted the transduction of genes to the postsynaptic inhibitory neurons (Figure 1C). DISECT has two anatomical requirements. This example of an M2-S1 top-down circuit illustrates the need for these requirements. First, DISECT requires direct projection from the M2 to the S1 postsynaptic region because AAV1-Cre needs to spread beyond the first-order synapse at S1 (Figure 2). Second, it needs no retrograde projections from S1 inhibitory neurons (INs) to M2 neurons (Figure 3). If the circuit has retrograde inhibitory projections, it causes false labeling of M2 projecting S1 INs as well as M2 input-recipient S1 INs.

To validate requirement 1, we performed anterograde axonal tracing from M2 to S1 (Figure 2A). We injected AAV1-CaMKII-ChrimsonR-tdTomato into the M2 (*n* = 3 mice). Three weeks after the injection, we confirmed tdTomato expression at the M2 injection site (Figures 2B, C) and M2 axons in S1 in coronal sections of the brain slices (Figures 2C, D). These axons were preferentially distributed in the superficial and deep layers of S1, which is the typical top-down cortical projection pattern (Felleman and Van Essen, 1991), as previously reported (Figures 2E, F; Manita et al., 2015).

To validate requirement 2, we investigated whether S1 INs project to M2. Although most INs in the neocortex have short-range projections, a few types of INs have long ranges (Tomioka et al., 2005). To identify whether S1 had INs with long-range projections to M2, we performed retrograde labeling of M2 projecting S1 neurons and calculated their fraction (Figure 3A). First, we injected the retrograde tracer CTB into the M2 and labeled M2 projecting S1 neurons (*n* = 2 mice). Five days after the injection, we observed the fluorescence of CTB at the M2 injection site (Figures 3B, C) and the CTB-labeled somata in S1 (Figures 3C, D) in brain slices. Next, we performed FISH on the slices to examine whether CTB-labeled S1 neurons expressed VGAT mRNA, a marker of INs. We found that only a small fraction of CTB-labeled S1 neurons expressed VGAT mRNA (Figures 3D, E). Quantitative analysis indicated that 1.7% (4 out of 240 neurons) of the CTB-labeled neurons expressed VGAT mRNA, and 98.3% (236/240) did not (Figure 3F). These results indicate that almost none of the M2 projecting S1 INs. Taken together, we conclude that DISECT is applicable to the M2-S1 top-down circuit.

### 3.2. DISECT visualizes M2-recipient S1 inhibitory neurons

We applied DISECT to study the properties of M2-input recipient S1 INs (Figure 4A). To transduce Cre recombinase into these neurons, we injected the AAV1-Cre into the M2 (Figure 4A). We performed a cocktail injection of Hoechst 33342 and AAV1-Cre to label the M2 injection sites. On the same day, we injected AAV1-hdlx-flex-GFP into the S1, which transduced GFP into the Cre + S1 inhibitory neurons (*n* = 5 mice). Four weeks after injection, we confirmed the fluorescence of Hoechst 33342 at the M2 injection site (Figures 4B, C), and GFP-labeled S1 neurons, which are M2-input recipient neurons, were located in all S1 layers (Figures 4C, D). To confirm whether the transduction of GFP by the hDlx enhancer was restricted to S1 INs, we performed FISH and confirmed that most GFP + neurons (95.4%, 83/87 neurons) expressed VGAT (Figures 4D–F). These results indicate that DISECT successfully labeled the M2 input recipient S1 INs.

### 3.3. Laminar profiles of M2-input recipient S1 INs

We performed a quantitative analysis of the laminar distribution of the GFP + INs. To analyze the laminar specificity of the GFP + INs, we statistically compared the fractions of GFP + S1 INs (i.e., M2-recipient INs) and VGAT + S1 INs (the total population of GABAergic INs) using binomial tests. We calculated laminar specific ratios of VGAT + INs (number of VGAT + INs in each cortical layer to the total number of VGAT + INs in all the layers) as the expected fractions of INs (Figure 5A). We also calculated the ratios of the GFP + neurons (number of GFP + INs in each cortical layer to the total number of GFP + neurons in all the layers). Statistical analysis revealed that the ratio of GFP + S1 INs in L4 was significantly smaller than the ratio of VGAT + neurons (Figure 5B). This result suggests that M2 axons tend to avoid making synaptic connections with S1 INs in the middle layers. We also found that the GFP + S1 neurons were preferentially located in the upper and deeper layers (Figure 5C, green box). Furthermore, we found that the laminar specificity of GFP + S1 neurons aligned with the laminar density of M2 axons shown in Figure 2F (magenta line), which was also lower in the middle layers. These results support the hypothesis that DISECT-labeled neurons receive presynaptic inputs from M2 axons.

### 3.4. Neuron types of M2-input recipient S1 INs

In the mouse cortex, PV, SST, and VIP are exclusive neurochemical markers of the INs (Rudy et al., 2011), and NPY is a marker of neurogliaform neurons (Schuman et al., 2019). We examined the expression of PV and SST by immunohistochemistry and VIP and NPY by FISH to determine which subtypes of INs were most dominant in M2-recipient S1 INs (Figure 6A).

Before investigating the coexpression of GFP and the markers, we validated our neurochemical marker staining results. To this end, we calculated the fraction of all INs that expressed each subtype-specific marker (i.e., VGAT + neurons) located in all cortical layers or in individual layer; we then compared the results with those of a previous study (Gonchar et al., 2008). First, we obtained the ratio of neuron density between each subtype and VGAT + neurons (as background INs; for more details, see Section “2.8. Data analysis” in Section “2. Materials and methods”). Our analysis indicated that the dominant subtypes were PV, SST, and VIP INs, in that order (Table 1). Second, we also confirmed the distribution of each subtype among the different cortical layers. We obtained the neuron density ratio for each cortical layer (Table 1; Figure 6B) and found similar laminar profiles of INs, represented by the absence of PV + neurons in cortical layer 1 and laminar specificity of VIP + neurons in superficial layers. These results are consistent with those of Gonchar et al. (2008) (Table 2).

**TABLE 1:** Total fractions of neurons expressing each neurochemical marker.

	Mean ratios of VGAT+ (GABA+) neurons			
Study	PV	SST	VIP	NPY
This study (S1)	0.30	0.28	0.14	0.24
Gonchar et al., 2008 (Primary visual cortex)	0.39	0.24	0.11	0.17

**TABLE 2:** Comparative fractions of neurons expressing specific neurochemical markers across cortical layers [Our study vs. Gonchar et al.’s (2008) study].

	Mean ratios of neurons in different layers					
	X (Y), where X: Fractions obtained in this study, Y: Fractions obtained in Gonchar et al.’s (2008) study					
Marker	L1	L2/3	L4	L5	L6	Total
PV	0 (0)	0.18 (0.20)	0.22 (0.27)	0.36 (0.29)	0.24 (0.23)	1.0 (1.0)
SST	0.004 (0.04)	0.24 (0.23)	0.11 (0.19)	0.33 (0.31)	0.32 (0.23)	1.0 (1.0)
VIP	0.06 (0.14)	0.47 (0.30)	0.17 (0.25)	0.16 (0.17)	0.14 (0.14)	1.0 (1.0)
NPY	0.06 (0.07)	0.38 (0.20)	0.11 (0.17)	0.14 (0.20)	0.29 (0.37)	1.0 (1.0)

After the validation, to analyze the fraction of neurons expressing each neurochemical marker in the GFP + neurons identified by DISECT, we counted the number of GFP + neurons coexpressing each neurochemical marker and calculated their ratio to the total number of GFP + neurons (Figure 6C). We statistically compared the fractions of the neurons coexpressing GFP + with each marker neuron, and each subtype shown in Figure 4B using binomial tests. The results showed that the fractions of GFP+ PV+ and GFP+ SST+ neurons were not significantly different from their expected fractions (Figure 6C). In contrast, the proportions of GFP+ VIP+ and GFP+ NPY+ neurons were significantly smaller than those of their expected fractions. These results indicate that M2-input recipient S1 neurons preferentially express PV and SST rather than VIP or NPY. The laminar profile of each subtype was analyzed using the same method (Figure 6D; Supplementary Table 1). In all subtypes, we confirmed that the fraction of GFP + neurons in the S1 L4 was smaller than the expected fractions, which is consistent with the results shown in Figure 5B. However, the fraction of GFP+ PV+ coexpressing neurons was significantly smaller in L2/3 and significantly larger in L6 than in their expected fractions. We also discovered that the fraction of GFP+ SST+ neuron coexpression in L1 was significantly larger than its expected fraction. We did not find any significant differences in the laminar profiles of GFP+, VIP+, or GFP+ NPY+ neuron coexpression. These results suggest that the M2-input recipient S1 + INs preferentially expressed PV in the deep layers and SST in the superficial layers. Taken together, we identified the subtype specificity and laminar profiles of M2 top-down input-recipient S1 INs using DISECT (Figure 6E).

## 4. Discussion

We developed DISECT, an efficient method for tracing postsynaptic IN, by combining conventional AAV-mediated anterograde transsynaptic tracing with Dlx enhancer-restricted gene expression. Using DISECT, we labeled M2 top-down input-recipient postsynaptic INs and revealed neuron subtypes and their laminar profiles. We found that almost all DISECT-labeled neurons expressed VGAT mRNA (Figure 4F) and that the neurons labeled with PV and SST were preferentially located in the deeper and upper cortical layers (Figure 6D), respectively. The laminar specificity of the DISECT-labeled neurons corresponded to that of the M2 top-down projection pattern (Figure 5C). These results indicate that DISECT can be used to visualize subtype-dependent laminar profiles of postsynaptic INs.

### 4.1. Advantages compared with conventional AAV-mediated transsynaptic tracing

Dlx enhancer-restricted Interneuron-SpECific transsynaptic Tracing succeeded in greatly simplifying conventional AAV-mediated transsynaptic tracing. This simplification reduces the experimental procedures and costs, increases efficiency, and extends the range of applications, as discussed below.

First, DISECT simplifies the *post hoc* analysis for subtype determination of INs by not labeling excitatory neurons, which has two advantages. One advantage of not labeling excitatory neurons, including larger fractions of retrogradely-projecting neurons, is prevention of false labeling and disturbing the *post hoc* analysis. Another advantage is that the neurons labeled by DISECT can be clearly identified as GABAergic INs without any additional histological procedures and analyses. This observation allowed us to instantly quantify the subtype specificity of the postsynaptic INs (Figure 6D). For example, fractions of labeled neurons that are smaller or larger than the expected total fractions indicate lower or higher preferences for target INs, respectively.

Second, DISECT simply requires wild-type mice and commercially available viral vectors, but not the generation of transgenic animals (e.g., breeding) or viral vectors (e.g., vector purification). This can accelerate the experiments and contribute to reducing costs and the number of animals.

Third, DISECT ensures higher applicability in a variety of experiments that require multiple intersectional systems. Combinations of orthogonal intersectional systems is necessary in various experimental procedures such as the conditional knockout of specific genes, optogenetic and chemogenetic manipulations, and activity-dependent labeling at the same time. However, the number of the available systems is currently limited to three (Fenno et al., 2020). The conventional tracing method which requires two major intersectional systems (Cre/cDIO and Flp/fDIO) would be difficult to be incorporated into the experiments because only one system (vCre/vcDIO) is left for the other procedures. Because DISECT requires only one of the systems, this will allow a variety of experimental methods to be used.

### 4.2. Subtype specificity and laminar profiles of M2-input recipient INs

Using DISECT, we found the IN subtype specificity of projections from M2, which target PV+ or SST+ rather than VIP+ or NPY+ (Figure 6C). Previous studies have reported subtype-specific top-down projections (Schneider et al., 2014; Zhang et al., 2014; Ma et al., 2021). For example, projections from the cingulate cortex to V1 preferentially target VIP+ neurons, resulting in disinhibitory effects on V1 activity. Projections from M2 to A1 preferentially target PV+ neurons and suppress auditory responses. We also found the subtype-dependent laminar profiles of the INs, which are difficult to describe using conventional anterograde approaches (Figures 6D, E). Our results showed that the M2 input-recipient PV + INs were predominantly distributed in the deep layers and less so in the superficial layers, thereby avoiding the middle layers. The laminar specificity of M2 top-down projections is different from that of other projections, i.e., S2 top-down projections to S1 target L2/3 PV + INs (Naskar et al., 2021). Together, these differences in the top-down projection recipients of the IN subtype and their laminar specificity would reflect different forms of sensory processing, depending on the modality.

### 4.3. Technical limitations

Although DISECT is a simple and efficient tracing tool, the following three points should be considered.

First, we considered the false labeling of retrogradely-M2-projecting S1 INs because retrograde transport of AAV1-Cre can also occur in INs. If the AAV1-Cre injected presynaptic site receives long-range inhibitory projections from a postsynaptic region, DISECT will result in false labeling of projecting INs as well as recipient INs in that region. Previous studies have reported a limited number of INs with projection distances longer than 2.0 mm in the cortical regions (Tomioka et al., 2005; Rock et al., 2018; Ruff et al., 2022). To avoid false labeling, it is necessary to confirm in advance whether the fraction of retrogradely-projecting INs in a targeted postsynaptic circuit is small enough to be ignored (Figure 3F).

Second, we considered subtype-dependent differences in *trans-*synaptic transport efficiency, which led to differences in comparisons between subtypes. A previous study confirmed that transsynaptic tracing methods can label both excitatory and inhibitory neurons (Zingg et al., 2020). However, the study also confirmed that postsynaptic neurons targeted by cholinergic projections cannot be labeled using AAV1-mediated methods. This result suggests potential differences in the labeling efficiency between IN subtypes. To clarify the anatomical results more precisely, comprehensive observations by multiple methods using retrograde or anterograde approaches, such as retrograde transsynaptic tracing using rabies viral vectors, channelrhodopsin-2 (ChR2)-assisted circuit mapping (CRACM), or anterograde transsynaptic tracing using other viral vectors (Li et al., 2021; Tsai et al., 2022), would be helpful.

Third, we examined the specificity of Dlx enhancers to IN subtypes. The enhancers used in DISECT—mDlx and hDlx—are gene sequences designed for gene transduction to universally target cortical INs. Previous studies have confirmed that there is little difference in the labeling efficiency between the major subtypes, including PV+, SST+, and VIP+ INs (Dimidschstein et al., 2016). However, a study using mGAD65 for gene transduction into INs have reported significantly higher transduction efficiency than mDlx in some subtypes, such as chandelier neurons (Hoshino et al., 2021). Therefore, the tracing results for some subtypes should be carefully interpreted in terms of enhancer specificity. Combinations of DISECT, other tracing methods, and functional recordings (e.g., optical or electrophysiology) with neural manipulations are crucial for further circuit-by-circuit clarification of the top-down-input-recipient inhibitory circuitry.

## 5. Summary

Dlx enhancer-restricted Interneuron-SpECific transsynaptic Tracing can be applied not only to label postsynaptic inhibitory neurons with fluorescent proteins but also to gene transduction of opsin and GECI for physiological experiments and more detailed subtype classification by neural morphology and transcriptome analysis (Gouwens et al., 2020; Scala et al., 2020). These experimental approaches will also allow us to characterize the anatomical, functional, and genetic properties of postsynaptic INs. DISECT enables visualization and classification of the precise properties of postsynaptic INs, which are difficult to study using conventional approaches. Further understanding of postsynaptic INs would rule out diverse projections in the brain.

## Data availability statement

The raw data supporting the conclusions of this article will be made available by the authors, without undue reservation.

## Ethics statement

The animal study was approved by the Animal Experiment Committee at RIKEN. The study was conducted in accordance with the local legislation and institutional requirements.

## Author contributions

YA and MM conceived the study and drafted the manuscript. YA designed the experiments and analyzed the data. YA and MO performed the histological experiments. YO, CM, KeK, SK, and KaK produced the AAV vectors. YA, YO, YS, HU, and MM revised the manuscript. All authors have read and approved the final version of the manuscript.
