# Supplementary material for: Anatomical identification of a corticocortical top-down recipient inhibitory circuitry by enhancer-restricted transsynaptic tracing
Source: Front Neural Circuits. 2023 Aug 30;17:1245097. doi: 10.3389/fncir.2023.1245097 (PMC10502327; doi:10.3389/fncir.2023.1245097)
Supplement: Supplementary file 1 [file Table_1.DOCX]

Supplementary Material

# Supplementary Figures and Tables

## Supplementary Tables

**Supplementary Table 1. Results of a binomial test shown in Figure 6D**

| **Markers** | **Layers** | **Total number of neurons** | **Number of neurons** | **Expected fractions** | **Obtained fractions** | **p-value** |
| --- | --- | --- | --- | --- | --- | --- |
| **PV** | **L1** | 69 | 0 | 0 | 0 | 1.0 |
|  | **L2/3** |  | 4 | 0.18 | 0.058 | 0.0066 |
|  | **L4** |  | 4 | 0.22 | 0.058 | 0.00040 |
|  | **L5** |  | 33 | 0.36 | 0.48 | 0.059 |
|  | **L6** |  | 28 | 0.24 | 0.41 | 0.0018 |
| **SST** | **L1** | 65 | 4 | 0.004 | 0.058 | 0.00030 |
|  | **L2/3** |  | 12 | 0.244 | 0.17 | 0.38 |
|  | **L4** |  | 1 | 0.11 | 0.014 | 0.0090 |
|  | **L5** |  | 24 | 0.33 | 0.36 | 0.36 |
|  | **L6** |  | 23 | 0.32 | 0.33 | 0.50 |
| **VIP** | **L1** | 8 | 0 | 0.06 | 0 | 1.0 |
|  | **L2/3** |  | 4 | 0.47 | 0.50 | 1.0 |
|  | **L4** |  | 1 | 0.17 | 0.13 | 1.0 |
|  | **L5** |  | 1 | 0.16 | 0.13 | 1.0 |
|  | **L6** |  | 2 | 0.14 | 0.25 | 0.30 |
| **NPY** | **L1** | 30 | 3 | 0.06 | 0.10 | 0.44 |
|  | **L2/3** |  | 10 | 0.38 | 0.33 | 0.70 |
|  | **L4** |  | 2 | 0.11 | 0.067 | 0.58 |
|  | **L5** |  | 7 | 0.14 | 0.23 | 0.18 |
|  | **L6** |  | 8 | 0.29 | 0.27 | 0.84 |
